# Evaluation of Elastin-Like Polypeptides for Tumor Targeted Delivery of Doxorubicin to Glioblastoma

**DOI:** 10.3390/molecules24183242

**Published:** 2019-09-06

**Authors:** Sonja Dragojevic, Rebecca Mackey, Drazen Raucher

**Affiliations:** Department of Cell and Molecular Biology, University of Mississippi Medical Center, 2500 North State Street, Jackson, MS 39216, USA (S.D.) (R.M.)

**Keywords:** elastin-like polypeptide, drug delivery, doxorubicin, glioblastoma multiforme

## Abstract

To increase treatment efficiency for glioblastoma, we have developed a system to selectively deliver chemotherapeutic doxorubicin (Dox) to Glioblastoma (GBM) tumors. This carrier is based on elastin-like polypeptide (ELP), which is soluble at physiological temperatures but undergoes a phase transition and accumulates at tumor sites with externally applied, mild (40–41 °C) hyperthermia. The CPP-ELP-Dox conjugate consists of a cell penetrating peptide (CPP), which facilitates transcytosis through the blood brain barrier and cell entry, and a 6-maleimidocaproyl hydrazone derivative of doxorubicin at the C-terminus of ELP. The acid-sensitive hydrazone linker ensures release of Dox in the lysosomes/endosomes after cellular uptake of the drug conjugate. We have shown that CPP-ELP-Dox effectively inhibits cell proliferation in three GBM cell lines. Both the free drug and CPP-ELP-Dox conjugate exhibited similar in vitro cytotoxicity, although their subcellular localization was considerably different. The Dox conjugate was mainly dispersed in the cytoplasm, while free drug had partial nuclear accumulation in addition to cytoplasmic distribution. The intracellular Dox concentration was increased in the CPP-ELP-Dox cells compared to that in the cells treated with free Dox, which positively correlates with cytotoxic activity. In summary, our findings demonstrate that CPP-ELP-Dox effectively kills GBM cells. Development of such a drug carrier has the potential to greatly improve current therapeutic approaches for GBM by increasing the specificity and efficacy of treatment and reducing cytotoxicity in normal tissues.

## 1. Introduction

Glioblastoma (GBM) ranks among the most common, aggressive, and least curable cancers due to a strong tendency for intracranial dissemination, high proliferation potential, and inherent tumor resistance to radiation or chemotherapy [1,2,3]. Standard of care for GBM patients is surgical removal of the tumor followed by chemotherapy with alkylating agent Temozolomide (TMZ) and radiation. The lack of favorable therapeutic outcomes can be ascribed to immense genetic instability resulting in a complex intra-and inter-individual heterogeneity [4], as well as adverse nonspecific treatment effects in normal brain tissue and other organs. Additionally, failure of GBM therapies is also attributed to poor or inadequate drug penetration through the blood brain barrier (BBB) or blood brain tumor barrier (BBTB). Despite the development of novel therapeutic approaches utilizing a wide variety of drug delivery systems in conjunction with other chemical and physical systems in tumor targeting or BBB disruption, the overall treatment efficacy and patient survival rates remain low. To address this problem, many drug delivery approaches for GBM treatment were developed. Two of these approaches include chemical disruption of the BBB by mannitol, which hyper-osmotically shrinks endothelial cells, providing space for large molecules to passively diffuse across the BBB, and focused ultrasound (FUS), used for a non-invasive physical approach to BBB disruption. Moreover, researchers have used receptor-mediated endocytosis, exploiting the natural receptor-ligand interactions that take place at the BBB, utilizing them for active transport of macromolecules. [5,6,7,8]. However, all of these treatments have limited effectiveness. New approaches for targeted deliveries are needed. 

Anthracycline antibiotic Doxorubicin is one of the most common chemotherapeutics used in the treatment of various solid and blood cancers [9,10]. Although Doxorubicin efficiently kills glioblastoma cells in vitro, its use for the treatment of brain tumors is limited due to poor penetration of the drug through the BBB and severe side effects in normal tissue, including dose-limiting cardiotoxicity. Previously, researchers have developed various formulations such as liposomes, nanoparticles, and polymer conjugates to improve transport of Doxorubicin through the BBB and delivery to glioblastoma tumors [11,12]. Additionally, complementary approaches, which include ultrasound, pH difference, and application of heat, have been used to disrupt the BBB [13,14,15].

In addition to approaches focused on disrupting the BBB, targeted approaches, which are based on macromolecular properties of the carriers, have been developed. These macromolecular carriers are designed to increase the solubility of small molecule drugs and enhance passive drug accumulation in tumors due to a unique tumor vasculature and poor lymphatic clearance [16].

A passive targeting approach was used to deliver a Doxorubicin derivative prodrug, doxorubicin (6-maleimidocaproyl) hydrazone (DOXO-EMCH). DOXO-EMCH, which was specifically designed to selectively bind to the cysteine-34 position of circulating serum albumin [17,18,19] and therefore passively accumulate in the tumors because of enhanced retention and permeability effect [16]. Marrero et al. used DOXO-EMCH (also known as Aldoxo) in a preclinical study of in vivo efficacy in an orthotopic GBM xenograft model. In vivo experiments have shown that DOXO-EMCH reduces tumor size by 10-fold and 8-fold for control and free Dox-treated animals, respectively. These preclinical studies prompted initiation of clinical trials. Although Phase 2 clinical trials with DOXO-EMCH showed some promising results, clinical trials with DOXO-EMCH never reached Phase 3. To improve the targeting approach employed by DOXO-EMCH, we have developed a thermoresponsive drug delivery system, which, in addition to passive targeting, employs an active targeting approach. This drug delivery system is based on elastin-like polypeptide (ELP), which is soluble at physiological temperatures but undergoes a phase transition and accumulates at tumor sites with externally applied, mild (40–41 °C) hyperthermia. We have modified the ELP sequence by including three cysteine residues at the C-terminus, which allows selective conjugation of DOXO-EMCH to the ELP biopolymer. Since ELP is thermally responsive, doxorubicin drug delivery by ELP will be enhanced in the tumor site when tumors are exposed to hyperthermia generated by high-intensity focused ultrasound. Furthermore, the acid-sensitive hydrazone linker of DOXO-EMCH ensures the release of Dox in lysosomes/endosomes after cellular uptake of the drug conjugate.

Cell-penetrating peptides have attracted great interest for their ability to translocate across the cellular plasma membrane and overcome such drug delivery transport barriers as those of the tumor microvessel walls and BBB [20]. Therefore, to enhance intratumoral uptake and cell entry, the ELP-DOXO conjugate was modified with an additional moiety, a cell penetrating peptide (CPP) [21]. In this study, we have used SynB1, a CPP which is derived from antimicrobial peptides known as protegrins, that are recognized to mediate cellular uptake of cargo via endocytotic mechanisms [22,23]. Furthermore, we have previously shown that SynB1-ELP can efficiently penetrate the BBB in a rat glioma model [24]. 

**Figure 1:**
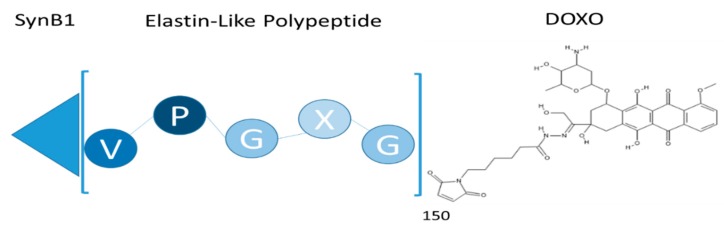
Elastin like polypeptide: Conjugate structure is composed of the cell penetrating peptide (SynB1), elastin-like polypeptide (ELP), and DOXO-EMCH (Doxo). MW = 60 kDa.

The purpose of this study was to examine the potential of SynB1-ELP-DOXO (Figure 1) to effectively inhibit glioblastoma cells and to demonstrate its potential for future use in preclinical animal studies. We showed that SynB1-ELP-DOXO kills cells effectively when compared to free Dox in three GBM cell lines (D54, GBM6, and U251-MG). To investigate the mechanism of action of SynB1-ELP-DOXO, we conducted uptake assays, apoptosis assays and cell cycle distribution assays using flow cytometry. Data from the uptake assays were used to compare the cytotoxicity of SynB1-ELP-DOXO with the amount of cellular uptake and protein association within and between different GBM cell lines. A positive correlation between the uptake and cellular killing was observed in all three GBM cell lines. To examine the mechanism of action of SynB1-ELP-DOXO and compare it to that of free Dox, we performed cell cycle distribution and apoptosis experiments. There was a noticeable difference in the cell cycle arrest between the cell lines. Both D54 and GBM had 60% of cells arrested in G2/M phase; while U251-MG had 90% of total cell population arrested in G2/M phase. The most sensitive cell line to any of the treatments used in the study was U251-MG. This study demonstrates that SynB1-ELP can be used as a doxorubicin carrier, efficiently killing glioblastoma cells in vitro, and it provides initial proof of principle for the use of SynB1-ELP-DOXO in future preclinical studies.

## 2. Results

### 2.1. Cytotoxicity of SynB1-ELP-DOXO and Free Doxorubicin 

**Figure 2:**
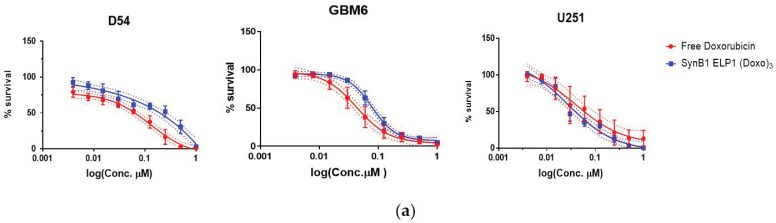

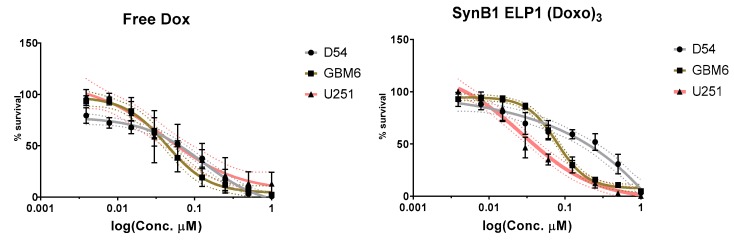
Cytotoxicity effects of free Doxorubicin and SynB1-ELP1-DOXO. D54, GBM6, and U251-MG cells were exposed to varying concentrations of SynB1-ELP1-DOXO, or free Dox for 72 h at 37 °C, and the number of viable cells was estimated with CellTiter-Glo^®^ assay. The data represent an average of at least three experiments (error bars represent S.E.M.). (**a**) Comparison of free Dox and SynB1-E1-DOXO cytotoxicity within cell lines. (**b**) Comparison of free Dox and SynB1-E1-DOXO cytotoxicity effect between cell lines.

SynB1-ELP-DOXO conjugates were compared to free doxorubicin for their ability to inhibit proliferation of glioblastoma cells. Cells were treated for 72 h with SynB1-ELP-DOXO or free doxorubicin, the number of viable cells was determined, and survival percentage was calculated with respect to untreated cells. The cell viability data were fitted to sigmoidal dose-response curves to determine IC50 values (Figure 2). All three glioblastoma cell lines have an IC50 within the nano-molar concentration range for both free Dox and SynB1-ELP-DOXO treatments (Table 1). U251-MG cell line was the most sensitive to the treatment, demonstrating 2.8 times higher sensitivity to SynB1-ELP-DOXO-EMCH treatment when compared to GBM6 and D54 cells lines. Similarly, U251-MG cell line was also most sensitive to free doxorubicin treatment which was 1.8 times more cytotoxic to U251-MG when compared to other cell lines. 

### 2.2. Cellular Association/Uptake of SynB1-ELP-DOXO and Free Doxorubicin

**Figure 3:**
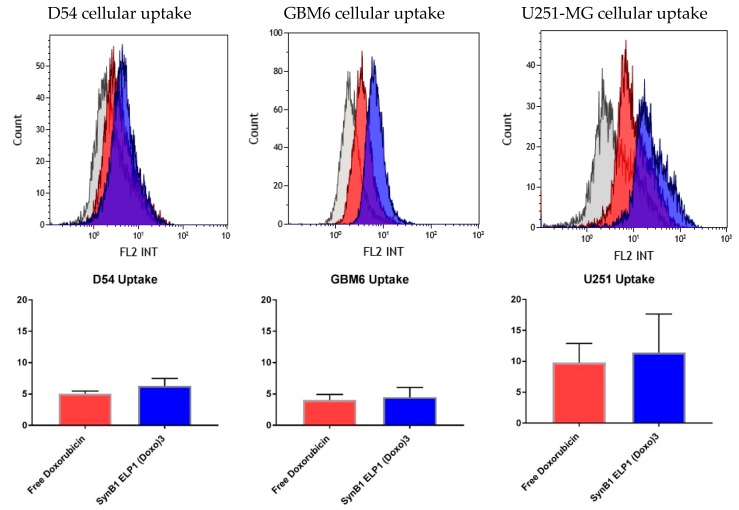
The upper panel shows representative cellular uptake histograms for D54, GBM6, and U251-MG cell lines. The lower panel represents average geometric mean of uptake for three independent experiments. Y-axis represents a relative fluorescence unit. Error bars are SEM.

To compare cellular association/uptake of SynB1-ELP-DOXO and free doxorubicin, GBM cell lines D54, GBM6, and U251-MG were incubated with 125 nM SynB1-ELP-DOXO or free Dox for 24 h. Representative flow cytometry histograms showing cell auto-fluorescence (gray), free doxorubicin (red) and uptake of SynB1-ELP-DOXO–EMCH (blue) are shown in Figure 3. Histograms represent total cell fluorescence, which include contribution from both extracellular and intracellular doxorubicin.

### 2.3. Cellular Localization of SynB1-ELP-DOXO and Free Doxorubicin 

**Figure 4:**
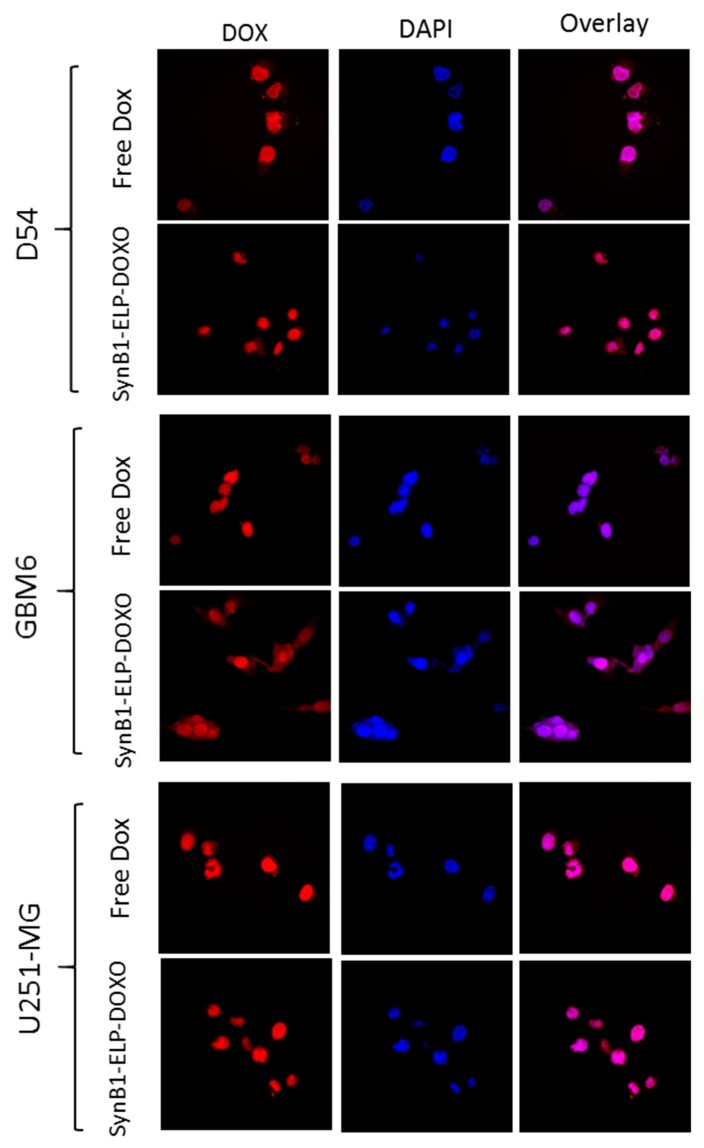
Comparison of intracellular localization of free Doxorubicin, and SynB1-ELP-DOXO in D54, GBM6, and U251-MG. Representative images of Dox fluorescence (red); nuclei stained with DAPI (blue); the overlay (purple) represents regions where doxorubicin has entered the nucleus. Any difference in image intensity between cells treated with free Dox or SynB1-ELP-DOXO is qualitative only and does not represent the actual difference in Dox concentration in the cells.

Cellular localization of doxorubicin delivered by SynB1-ELP-DOXO and with free doxorubicin was examined by confocal fluorescence microscopy. Cells were incubated with 25 µM Dox equivalent concentration of SynB1-ELP-DOXO or free Dox for 2 h. As shown in Figure 4, Free Dox was predominately localized in the nuclei in all of the three cell lines (Figure 4) while SynB1-ELP-DOXO had mainly cytoplasmic localization in the perinuclear region as well as presence in the nuclei.

### 2.4. Apoptosis Assay 

**Figure 5:**
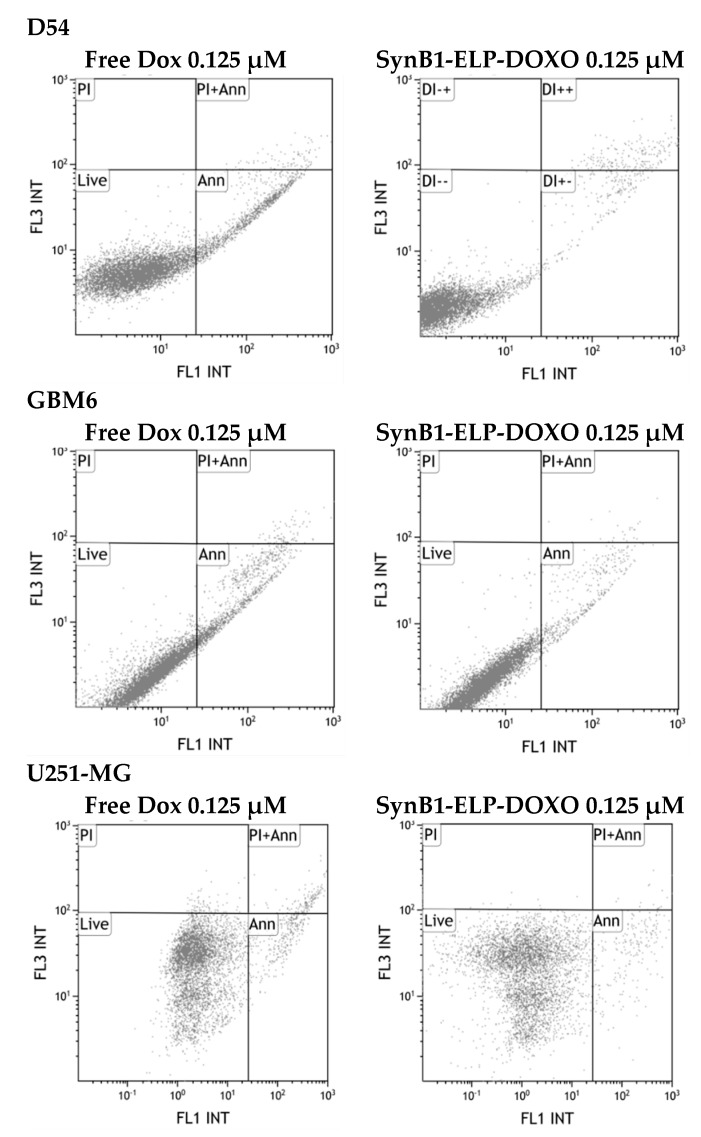
Induction of apoptosis by SynB1-ELP-DOXO in GBM cell lines. Cells were treated with 125 nM SynB1-ELP-DOXO or free Dox and analyzed 24 h after the treatment. Raw data for a representative experiment is shown.

Annexin assay was performed to evaluate the induction of apoptosis by SynB1-ELP-DOXO in GBM cell lines. Cells were gated based on double staining with PI and Annexin Alexa 488 nm. We have quantified the percent of apoptotic cells (Table 2) after incubating with free Doxorubicin and SynB1-ELP-DOXO for 24 h. The cell line with the highest percentage of apoptotic cells was GBM6 for both treatments, while U251-MG and D54 showed a reduced percentage of apoptotic cells with SynB1-ELP-DOXO and free Dox. Representative histograms of Annexin assay are shown in Figure 5.

### 2.5. Effect of SynB1 ELP DOXO and Free Doxorubicin on Cell Cycle

**Figure 6:**
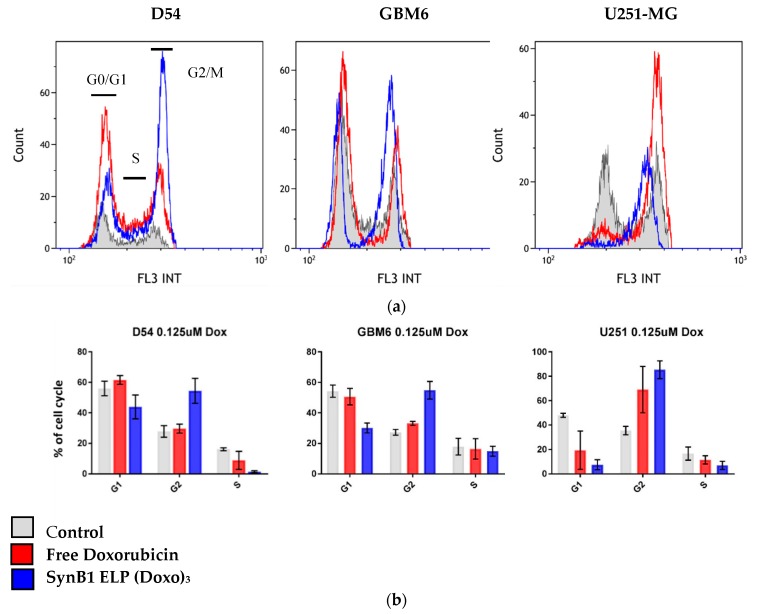
Cell cycle distribution. Cells were treated with 125 nM SynB1-ELP-DOXO or free Dox and analyzed 24 hours after the treatment. Raw data for a representative experiment is shown (**a**) and an average of three independent experiments is shown (**b**). Summary of results presented in B were determined by analysis of 10,000 cells per sample, and results represent the mean +/− SEM.

To compare cell cycle distribution, cells were treated with SynB1-ELP-DOXO or free Dox for 24 h at 37 °C at a concentration of 125 nM. As shown in Figure 6, SynB1-ELP-DOXO treatment exhibited a preferential block of G2/M phase of cell cycle in all three cell lines at the expense of S phase. The most significant increase in cell accumulation in G2/M (90% when compared to control 40%) was observed in U251-MG cell line, with respect to G0/G1 and S phase. D54 and GBM6 cells showed response toward SynB1-ELP-DOXO treatment similar to U251-MG, although the arrest toward G2/M phase was less significant. In D54 and GBM6, cells treated with free Dox showed an increase in percentage of cells in G1 and G2. SynbB1-ELP-DOXO treatment resulted in a similar response but with a more pronounced effect. GBM6 cells responded to free Dox with an increase in cell number in G0/G1 and G2/M phase.

## 3. Discussion

The prognosis for patients diagnosed with glioblastoma multiforme (GBM) is often dismal. Despite ongoing research efforts, therapeutic targeting, and identification of well-defined molecular markers, curative treatment options for GBM patients are still deficient [25]. The overall median survival rate of patients diagnosed with GBM is 15–23 months with less than 6% attaining five-year survival, which accounts for the lowest long-term survival of all brain tumor patients [26]. 

Doxorubicin is one of the potent drugs that has been successfully used for the treatment of many solid tumors, hematological malignancies, and cancers of the central nervous system. A study by Veringa et al. in 2013, an in vitro drug screen on primary glioma cells established from patient samples in pediatric high-grade gliomas (pHGG), reveals a high in vitro cytotoxicity of several commonly used chemotherapeutics including doxorubicin [27]. 

In vivo studies with doxorubicin derivative DOXO-EMCH, which binds to the Cys34 position of human serum albumin (Aldoxo-HSA), circumventing systemic toxicity (Kratz et al., 2000), demonstrated superior response against GBM tumors in mice when compared to free Dox treatment. While Aldoxo-HSA tumor targeting relies only on passive targeting due to EPR effect, the ELP-based thermally targeted drug delivery system can be actively targeted to the tumor site by application of hyperthermia (Raucher et al., 2018) while also benefiting from passive accumulation. This result was confirmed in our previous work where we have demonstrated that genetically modified ELP with a peptide inhibitor of c-Myc can be effectively delivered to and reduce brain tumors in a rat glioma model [24]. 

The purpose of this study was to extend this approach to small molecule drugs, such as doxorubicin, and evaluate if Dox delivered by SynB1-ELP is effective in the treatment of GBM cell lines.

Due to the large molecular weight of ~60 kDa, the ELP conjugate may not be able to pass the BBB and diffuse through the cell’s plasma membrane. To overcome this limitation, we added a cell penetrating peptide which enabled CPP-ELP drug carriers to accumulate in brain tumors in vivo (GBM in rats) [24] and enter the cells through adsorptive endocytosis [28]. Although adsorptive endocytosis uptake was less efficient in comparison to free diffusion of small and hydrophobic molecules, such as free Doxorubicin, SynB1-ELP-DOXO showed comparable cell killing of glioblastoma cells when compared to free doxorubicin. 

In this study, we have shown that SynB1-ELP-DOXO effectively inhibits cell proliferation in three GBM cell lines; GBM6, D54, and U251-MG with IC50 values in the nanomolar range, specifically 40 nM for GBM6 and U251-MG, and 120 nM for D54. Both the free drug and SynB1-ELP-DOXO exhibited similar in vitro cytotoxicity, although their subcellular localization was considerably different. SynB1-ELP-DOXO showed slightly higher toxicity (IC50 = 27 nM) than free Dox (IC50 = 40 nM) in U251-MG cell line. In D54 and GBM6, free doxorubicin had marginally better efficiency in killing cells than SynB1-ELP-DOXO. 

The difference in killing efficiency between the cell lines correlates directly to the difference in the level of cellular uptake of SynB1-ELP-DOXO. U251-MG had the highest uptake rate and the lowest IC50 values, while D54 and GBM6 had lower uptake rate, with higher IC50 when compared to U251-MG values under SynB1-ELP-DOXO treatment. However, the difference in toxicity between SynB1-ELP-DOXO and free Dox might not be the same in vivo, due to drug penetration and distribution in the tumor and more complex interactions of the drug within the unique GBM tumor microenvironment. 

Although Dox delivered by SynB1-ELP has a similar toxicity as a free Dox, it is important to note that treatment with SynB1-ELP delivered Dox can be targeted specifically to the tumor site and therefore it will have significantly less nonspecific toxicity in normal tissue. 

The cellular internalization of SynB1-ELP1-DOXO through adsorptive endocytosis, results in the acid-sensitive drug being exposed to the low pH environment of the endosomes/lysosomes, where it can be cleaved and released inside the cell. The cellular site of action of Dox is mainly in the nucleus, where it has been shown to intercalate into the DNA [29], while previous studies have shown that DOXO-EMCH-protein conjugates will localize in the cytoplasm and perinuclear areas. Our confocal localization experiments show that SynB1-ELP-DOXO has perinuclear localization, while free Dox was accumulated primarily in the nucleus. This suggests a different mechanism of action for killing the GBM cells other than just DNA intercalation, since the construct was mainly distributed in cytoplasm. GBM6 cell line showed a primarily cytoplasmic distribution of SynB1-ELP-DOXO, which was also accompanied by the lowest uptake of Doxorubicin, which may indicate higher drug resistance, compared to D54 and U251-MG cell lines.

To investigate the mechanism of action of doxorubicin delivered by SynB1-ELP and compare to that of free Doxorubicin, we analyzed cell cycle distribution. One of the established mechanisms of Doxorubicin cell killing is by intercalating into the DNA and inhibiting Topoisomerase II, resulting in cell cycle arrest [30]. Cell cycle analysis of GBM cells treated with free Dox showed enhanced arrest of the cells in G1 phase, while there was increased population of cells in G2/M phase in cells treated with SynB1-ELP-DOXO, when compared to free Dox, with some notable differences between cell lines. U251-MG showed the expected cell cycle arrest in G2/M phase with almost 90% of the cell population upon treatment with SynB1-ELP-DOXO, consistent with that free Dox. Cell lines D54 and GBM6 had only 60% arrest in G2/M phase. A difference in cell cycle distribution between D54 and U251-MG cells can be explained by the variable response to DNA damage due to a difference in DNA damage repair mechanisms [31]. 

The mechanism by which SynB1-ELP-DOXO induces apoptosis was examined by Annexin V binding, which is used to detect phosphatidylserine, a marker of apoptosis when it is localized on outer leaflet of the plasma membrane. After 24 h of incubation, the total apoptosis percentage of cells treated with free Dox was 1.5% in GBM6, 6% in D54, and 7% in U251-MG cells. However, the total apoptosis rates of SynB1-ELP-DOXO treated cells were 5%, 12% and 5% in GBM6, D54, and U251-MG cells, respectively. These data confirm that SynB1-ELP-DOXO induced apoptosis more effectively, in spite of its decreased toxicity, indicating that free doxorubicin and SynB1-ELP-delivered doxorubicin have a different mechanism of action.

One of the potential problems in glioblastoma treatment is the development of drug resistance. If Dox resistance is developed, the SynB1-ELP-DOXO construct could be adapted using a different linker between ELP and the doxorubicin derivative. Instead of joining the doxorubicin derivative molecule to ELP with a hydrazone linker, as used in this study, a peptide linker containing the amino acid sequence GFLG can be used (CPP-ELP-GFLG-Dox). This sequence is enzymatically cleaved by Cathepsin B proteases and would yield a doxorubicin derivative that is not a substrate for P-gylcoprotein. We have shown previously that [32] CPP-ELP-GFLG-Dox successfully and efficiently killed doxorubicin-resistant breast cancer cell lines. Therefore, a similar approach can be studied in the treatment of doxorubicin-resistant GBM cell lines, and this will be a subject of future investigation.

## 4. Materials and Methods 

### 4.1. Polypeptide Expression and Purification

Previously designed and cloned SynB1 ELP1 [33] polypeptide was hyper- expressed in *E. coli* BLR (DE3) (Novagen, Madison, WI, USA) and purified by inverse thermal cycling [34].

### 4.2. Conjugation of DOXO-EMCH to Biopolymer 

Doxorubicin derivative (DOXO-EMCH) with acid-cleavable (6-maleimidocaproyl) hydrazone linker was synthesized as previously described by Kratz et al. (DOXO-EMCH, generously provided by Dr. F. Kratz, CytRx Pharmaceuticals, Freiburg, Germany). The DOXO-EMCH was then covalently linked to three cysteine residues on ELP by thiol-maleimide coupling. To prevent spontaneous formation of disulfide bonds and maximize efficiency of the drug labeling process, protein conjugation with DOXO-EMCH was done under the following conditions: SynB1-ELP1-(GGC)_3_ protein at a concentration of 100 µM was solubilized in 50 mM sodium hydrogen phosphate (Na_2_HPO_4_) elution buffer, pH = 7, with the addition of 10 fold molar excess (1 mM) of tris (2-carboxyethyl) phosphine (TCEP) at room temperature for 30 min. Subsequently, freshly prepared 800 µM DOXO-EMCH was added to the protein solution and left to incubate for another 30 min at room temperature in the dark, followed by O/N incubation at 4 °C and protected from light. Unreacted DOXO-EMCH was removed by inverse thermal cycling. Protein concentration and labeling efficiency was estimated by measuring absorbance at 280 nm and 495 nm, respectively. The protein-drug concentration was calculated as described [35].

(1) $$Protein=\;\frac{\left(Abs\;280\;\mathrm{nm}-Abs\;350\;\mathrm{nm}\right)\;}{\varepsilon_{protein}\;}$$

(2) $$Drug\;:Protein\;conjugate\;=\frac{Abs_{280\mathrm{nm}}-\left(0.713\;\times \;Abs_{495\mathrm{nm}}\right)}{9250\;M^{-1}{\mathrm{cm}}^{-1}}$$

### 4.3. Cell Lines 

D54, GBM6, and U251-MG human glioblastoma cell lines were purchased from ATCC (American Type Culture Collection). Cells were maintained and grown in Dulbecco’s modified Eagle’s minimum essential medium (DMEM) (Corning, Thermo Fisher Scientific, Waltham, MA, USA), supplemented with 10% fetal bovine serum (FBS) (Atlanta Biologicals, Lawrenceville, GA, USA) and 1% Penicillin/Streptomycin antibiotics (HyClone, Thermo Fisher Scientific).

### 4.4. Cytotoxicity Assay

Cell proliferation was determined by CellTiter-Glo^®^ luminescent cell viability assay. Briefly, cells were seeded in opaque-walled 96-well plate with a density of 1 × 10^3^ cells per well in triplicates. After overnight incubation, cells were treated with two-fold increasing concentrations of free doxorubicin, SynB1-ELP-DOXO for 72 h.

The results of CellTiter-Glo^®^assay are based on cell lysis and generation of a luminescent signal proportional to the amount of ATP present. The treated cells were than assayed per manufacturer’s instructions and luminescent signal that is proportional to the amount of ATP present and number of cells present in the cell culture was quantified on the plate reader (Synergy H4 Hybrid Reader, BioTek^®^ Instruments, Inc., Winooski, VT, USA). Cell survival was expressed as a percentage of the signal intensity normalized to that of untreated control cells. Cell survival data were analyzed using GraphPad Prism and dose response curves were analyzed and fitted to a sigmoidal four PL—sigmoidal function.

### 4.5. Apoptosis Assay

Apoptosis was measured by flow cytometry using Gallios Flow Cytometer (Beckman Coulter, Fullerton, CA, USA). Briefly, cells were seeded in transparent 6-well plates with density of 5 × 10^4^ cells per well for D54, and 1 × 10^5^ for GBM6 and U251-MG. After overnight incubation, cells were treated with either free Doxorubicin or SynB1-ELP-DOXO (62.5 nM). Etoposide at a final concentration of 500 µM was used as a positive control. After 24 h both floating and attached cells were harvested and stained with conjugated Alexa Annexin-V and Propidium iodide (PI). Forward versus side scatter gating was used to eliminate cell debris from the analysis, and a scatter plot of PI intensity versus Alexa Annexin V intensity was used to score live, apoptotic, and necrotic cells. The percentage of Annexin-V positive, apoptotic cells was expressed as an average of three experiments. 

### 4.6. Cell Cycle Analysis

D54, GBM6, and U251-MG cells were plated in 6-well plates at density of 1 × 10^5^ for D54 and 5 × 10^4^ for GBM6 and U251-MG. The following day, cells were treated with SynB1-ELP-DOXO or free doxorubicin for 24 h at 37 °C. After the treatment, the cells were rinsed with PBS, fixed with 3 mL ice-cold 70% ethanol for 30 min, rinsed with PBS again and resuspended in 500 μL PBS. To eliminate signal from RNA, we added RNase A (Sigma Aldrich, Saint Louis, MO, USA) to a final concentration of 750 µg/mL to the cell suspension for 5 min. Cells were then treated with 200 μg/mL of Propidium iodide (PI) (Sigma, St. Louis, MO, USA) for 30 min at room temperature. To evaluate the intensity of PI fluorescence, as a measure of DNA content, we used Gallios flow cytometer and analyzed the results with Kaluza software (Beckman Coulter). A plot of forward scatter vs. PI intensity was gated to remove cell debris and cell aggregates from the analysis. Fluorescence was measured for a sample of 10,000 cells using FL3 (laser ex. 488 nm, filter 620/30 nm), and histograms of cell number versus PI intensity were used to determine the percentage of cells in each phase of the cell cycle.

### 4.7. Fluorescence Microscopy

In order to compare intracellular localization of SynB1-ELP-DOXO and free doxorubicin, glioblastoma cells were plated on 22 mm^2^ cover slips at approximately 50% confluence, and then incubated at 37 °C for 24 h. Cells were then treated with SynB1-ELP-DOXO and free doxorubicin (normalized to 25 uM concentration of doxorubicin) and incubated for 2 h, washed with PBS, fixed with ice-cold methanol, stained with DAPI, mounted on slides, and imaged with a Nikon confocal microscope (Nikon C2+,Nikon Instruments Inc., Melville, NY, USA).

### 4.8. Cellular Uptake Assay

D54 (1.0 × 10^5^ cells/well), GBM6 and U251-MG (5.0 × 10^4^ cells/well) were plated in 6-well tissue culture plates, and incubated for 24 h at 37 °C. After overnight incubation, cells were exposed to a range of concentrations of free Dox or SynB1-ELP-DOXO for 24 h. To harvest the cells after the treatment, we used a non-enzymatic cell dissociation buffer, which does not degrade polypeptide attached to the cells. Forward versus side scatter gating was used to remove cell debris from the analysis and Dox fluorescence intensity (*n* = 10,000 cells) was measured using a Gallios flow cytometer and Kaluza software (Beckman Coulter). Fluorescence intensity was normalized to cellular auto-fluorescence. 

## 5. Conclusions

In summary, our findings demonstrate that SynB1-ELP-DOXO effectively kills GBM cells and has potential as a macromolecular drug carrier for the intracellular delivery of Doxorubicin. SynB1-ELP delivered Dox to the cell cytoplasm, arrested cell cycle in G2/M phase, and induced apoptosis resulting in inhibition of cell proliferation. This work provides initial proof of principle for the use of ELP, as a thermally targetable delivery system for doxorubicin in vitro, and these results encourage the future evaluation of the efficacy of ELP as a potential drug carrier in vivo for the treatment of glioblastoma. In addition, the ELP system is very versatile and can be utilized for delivery of other small molecule therapeutics, which are currently limited due to an inability to penetrate BBB or due to detrimental side effects. Further studies are necessary to evaluate efficiency of SynB1-ELP-DOXO delivery to the brain tumors, which could possibly be a successful strategy in the treatment of aggressive tumors, such as GBM, alone or in combination with current treatments. Continued development of this drug carrier has the potential to improve current therapy outcomes for GBM patients by increasing the specificity and efficacy of treatment and reducing cytotoxicity in normal tissues.

## Figures and Tables

**Table 1:** IC50 values of human glioblastoma cell lines.

Cell Line	IC50 µM (Free Dox)	IC50 µM (SynB1-ELP-DOXO)
D54	0.12750 ± 0.04328	0.09 ± 0.0012
GBM6	0.04309 ± 0.00962	0.07854 ± 0.00821
U251-MG	0.04030 ± 0.02585	0.02767 ± 0.01306

**Table 2:** Percentage of Apoptotic Cells. The percentage of Apoptotic Cells is shown as an average of three independent experiments. Summary of results were determined by analysis of 10,000 cells per sample, and results represent the mean +/− SEM.

Cell Line	% Apoptotic Cells Free Dox	% Apoptotic Cells SynB1-ELP-DOXO
D54	11.85 ± 2.41	2.90 ± 1.13
GBM6	14.11 ± 0.66	9.34 ± 5.04
U251-MG	5.46 ± 0.9	7.24 ± 1.83
